# Antifungal Efficacy of Amphotericin B in *Candida Albicans* Endocarditis Therapy: Systematic Review

**DOI:** 10.21470/1678-9741-2019-0159

**Published:** 2020

**Authors:** Lucas Soares Bezerra, Janielli Assis da Silva, Marcelo Antônio Oliveira Santos-Veloso, Sandro Gonçalves de Lima, Ândrea Virgínia Chaves-Markman, Moacir Batista Jucá

**Affiliations:** 1 Centro Universitário Mauricio de Nassau, Recife, Brazil.; 2 Pós-graduação em Inovação Terapêutica (PPGIT), Centro de Biociências, Universidade Federal de Pernambuco, Recife, Brazil.; 3 Departamento de Cardiologia, Hospital das Clínicas da Universidade Federal de Pernambuco (HC/UFPE), Recife, Brazil.

**Keywords:** Amphotericin B, Antifungal Agents, Candida Albicans, Endocarditis, Mycoses, Survival Rate

## Abstract

**Introduction:**

Although it is the most common agent among the fungal causes of endocarditis, *Candida albicans* endocarditis is rare.

**Objective:**

To evaluate the efficacy of amphotericin B in the treatment of *C. albicans* endocarditis beyond a systematic review.

**Data search:**

Articles in English, Spanish and Portuguese, conducted in the following databases: MEDLINE, LILACS, IBECS and SciELO, in humans and published in the last 25 years.

**Study selection:**

Observational studies, clinical trials, and case series providing data on the amphotericin B use in patients with a *C. albicans* endocarditis diagnosis without age limitations.

**Data synthesis:**

From the initial search (n=79), 25 articles were fully evaluated, of which 19 were excluded for meeting one or more exclusion criteria, remaining five articles (two observational studies and three case series). Patients using amphotericin B demonstrated improvement in survival rates, and its main use was in association with the surgical method as well as with caspofungin association.

**Conclusion:**

Literature lacks evidence to conclude about efficacy and safety of amphotericin B in the treatment of fungal endocarditis. Randomized clinical trials are necessary to provide better evidence on the subject.

**Table:** 

Abbreviations, acronyms & symbols	
AHRQ	= Agency for Healthcare Research and Quality
AMB	= Amphotericin B
FE	= Fungal endocarditis
MIC	= Minimum inhibitory concentration
NIH	= National Institutes of Health

## INTRODUCTION

Fungal endocarditis (FE) is a rare disease, occurring mainly in patients with predisposing host conditions^[1]^, but with a propensity to be severe. *Candida* species are the most usual etiological agents, with *C. albicans* being the most common among them^[2-4]^. Infective endocarditis caused by *Candida* spp. is associated with a high mortality rate, between 30% and 80%^[5]^.

Besides the severity of this opportunistic infection, its diagnosis is difficult and also delayed, due to the low suspicion of the physician and the poor sensitivity of blood cultures^[2]^. Determining patients at high risk of FE is not simple considering the lack of information in medical literature about the clinical features and therapy^[1]^. Predisposing conditions are previous surgery, intravenous drug addiction, underlying heart disease, indwelling foreign bodies (catheters, prosthetic valves or pacemakers), immunosuppression, post-transplantation, post-chemotherapy, prolonged use of broad-spectrum antibiotics, HIV, and chronic underlying diseases as diabetes mellitus^[1,6-8]^.

The first-line drug for *C. albicans* endocarditis is amphotericin B (AMB), often followed by fluconazole because of frequent relapses^[6]^. AMB deoxycholate therapy acts by binding to the ergosterol of fungal membranes, creating channels through which vital molecules leak from the cells, leading to cellular death^[9]^. However, *C. albicans* is capable of biofilm formation, which serves as a barrier to block penetration of antimicrobial agents and confers resistance to several antifungals, which can decrease the activity of antifungals that target ergosterol such as amphotericin^[10,11]^.

Surgical intervention is considered the gold standard in addition to pharmacological therapy^[12]^, but in some cases a good response has been achieved only with drug therapy, especially AMB^[9,13]^. These cases include patients with minor vegetations found and high risk of perioperative death, whose pharmacological therapy should be first-line treatment^[14,15]^. Some studies demonstrate that surgical approach is not associated with increased survival rate compared to antifungal monotherapy with AMB^[6,14]^. Furthermore, among patients without surgical intervention, the history of relapses was not observed^[15]^. Therefore, antifungal monotherapy with AMB may be considered, especially in critically ill patients in whom surgery is not a viable option^[13]^.

Although some international guidelines recommend AMB as first-line drug in the treatment of FE, evidence regarding this topic is scarce and controversial. To our knowledge, this is the first systematic review on this subject.

### Objective

To evaluate the antifungal efficacy of amphotericin B in the treatment of *C. albicans* endocarditis; elucidate the drug efficacy, including its use as a complement or substitute for the surgical approach.

## METHODS

### Protocol and Registration

The methods used in this work followed the systematic review process derived from the PRISMA statement^[16]^. Details of the study protocol are registered on PROSPERO under registration number CRD42019106445.

### Eligibility Criteria

#### Types of studies

This paper covered observational studies, clinical trials, and case series published in the last 25 years in English, Spanish or Portuguese. Considering that this study aims to evaluate amphotericin B effects only in humans, experimental studies were not included.

#### Types of participants

Patients with clinical and mycological diagnosis of *C. albicans* endocarditis. No age restrictions were applied.

#### Types of interventions

The intervention consisted of the use of AMB alone or in association compared to placebo, another antifungal therapy and/or surgical treatment for FE.

#### Types of outcome measures

We considered as outcome measures: all-cause death, length of treatment, mycological cure (defined as negative blood culture after treatment), relapse of infection. In addition, the serum minimum inhibitory concentration (MIC) was evaluated as a variable.

### Information Sources

The following databases were consulted: MEDLINE, LILACS, IBECS and SciELO. Publications in English, Spanish or Portuguese were accessed. We considered only studies performed in humans and published in the last 25 years. The last search was run on January 2, 2019. The full electronic search strategy is available in Appendix 1.

### Study Selection

The eligibility assessment was executed independently and unblended standardized by two authors. Disagreements were resolved by a third author.

### Data Collection Process

Data extraction was made using a standard form. The form consisted of a pilot test in randomly selected included studies and adjusted accordingly. Disagreements were resolved by a third author.

### Data Items

The following data was extracted from full-text articles: (1) participant characteristics (including age, sex, number, associated comorbidities, and diagnostic method), (2) study design (including design, follow-up period, and type of intervention), and (3) outcomes (including mortality and survival rate).

### Risk of Bias in Individual Studies

To ascertain the validity and risk of bias, the reviewers determined the adequacy of data extraction, the blinding status of the study, the loss of follow-up and homogeneity of the examined samples. To help assess the quality of the studies, two different scales were used, according to the study design.

The quality of observational studies was assessed by the Agency for Healthcare Research and Quality (AHRQ) criteria for observational studies^[17]^. Acceptable quality was defined as a score of at least 50 points out of 100. The quality assessment is described in Table 1.

**Table 1:** Quality assessment for observational studies according to the AHRQ criteria.

Criteria	Weighted score points	Badiee et al.^[18]^, Iran, 2014	Melgar et al.^[19]^, USA, 1997
Study question	(0-2)	2	2
Study population	(0-8)	8	8
Comparability of subjects	(0-22)	9	6
Exposure or intervention	(0-11)	11	8
Outcome measure	(0-20)	20	15
Statistical analysis	(0-19)	12	17
Results	(0-8)	8	5
Discussion	(0-5)	5	5
Funding	(0-5)	5	0
TOTAL	(0-100)	80	66

The quality of the case series was assessed by the National Heart, Lung and Blood Institute (NIH) scale. An acceptable quality was defined as a minimum score of five yeses out of nine criteria, as shown in Table 2.

**Table 2:** Quality assessment for case series according to the NIH criteria.

Criteria		Stripeli et al.^[13]^, Grécia, 2008	Karatza et al.^[21]^, Grécia, 2008	Flanagan et al.^[20]^, Inglaterra, 1997
		Yes/No/Other^a^	Yes/No/Other^a^	Yes/No/Other^a^
1.	Was the study question or objective clearly stated?	Yes	Yes	Yes
2.	Was the study population clearly and completely described, including a case definition?	Yes	Yes	Yes
3.	Were the cases consecutive?	NR	NR	NR
4.	Were the subjects comparable?	Yes	Yes	No
5.	Was the intervention clearly described?	Yes	Yes	Yes
6.	Were the outcome measures clearly defined, valid, reliable, and consistently implemented across all study participants?	Yes	Yes	Yes
7.	Was the follow-up duration adequate?	Yes	No	NA
8.	Were the statistical methods well described?	NA	NA	NA
9.	Were the results well described?	Yes	Yes	Yes

a Other: CD=cannot determine; NA=not applicable; NR=not reported

### Summary Measures

The primary measure of treatment efficacy was the relative risk of declining mortality or increased survival rates.

### Additional Analysis

The sensitivity analysis was evaluated according to the quality components pointed in the AHRQ and NIH scales.

## RESULTS

**Fig. 1:**
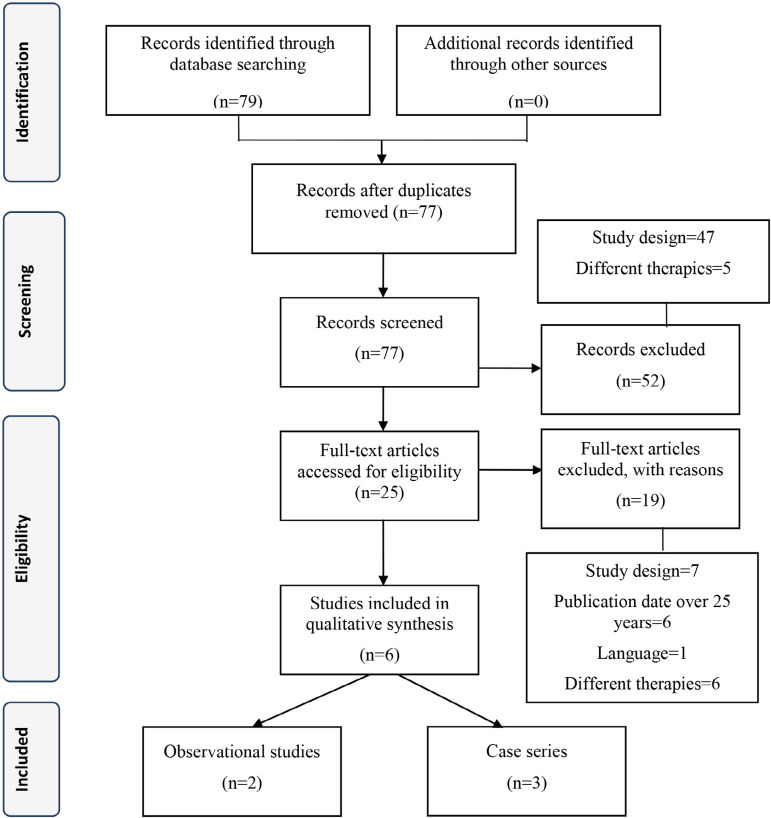
PRISMA flow diagram of studies.

From the initial search (n=79), 25 articles were fully reviewed, as demonstrated in Figure 1. From those, 19 were excluded: seven because of study design, six because the publication date was over 25 years, six because evaluated therapy did not include AMB, and one because of language restriction (Chinese). There were five articles that met the selection criteria: two observational studies and three case series. The methodological assessment of the studies is shown in Table 3.

**Table 3:** Summary of included studies evaluating the antifungal efficacy of AMB in the treatment of *C. albicans* endocarditis.

Source	No. of patients	Age range	Study design	Inclusion criteria	Follow-up (months)	Death rate (%)	Median blood sample MIC (µ/mL)	Median time to start antifungal treatment (days)	Negative blood culture after treatment (%)/time for negative result (days)	Relapse (%)/median time for relapse (months)
**Studies with adults**										
Badiee et al.^[18]^, 2014	2 (from 11)^a^	21-31 years	Cross-sectional	Infectious endocarditis patients after non-responsive antibacterial treatment, positive culture and transthoracic echocardiogram	12	0 (n=0)	0.375	NR	50/NR	NR
Melgar et al.^[19]^, 1997	9 (from 16)^a^	27-71 years	Case-control	Diagnosis of FE according to histological evidence, plus at least one positive culture (same fungus from the histological tissue)	54	11 (n=1)	NR	NR	NR	11/28
Flanagan et al.^[20]^, 1997	2 (from 3)^a^	74-76 years	Case series	Blood culture confirmed by post-mortem histology.	NR	100 (n=2)	0.275	6	NR	NR
**Studies with infants**										
Karatza et al.^[21]^, 2008	2	<1 month	Case series	Premature newborns diagnosed by clinical features, positive blood culture and transthoracic echocardiogram	NR	0 (n=0)	0.0195	19	50/3	NR
Stripeli F et al.^[13]^, 2007	2	2-3 months	Case series	Children diagnosed by clinical features, positive blood culture and echocardiogram	54	0 (n=0)	NR	7	50/4	0/NA

AMB=amphotericin B; EA=etiological agent; FE=fungal endocarditis; L-AMB=liposomal amphotericin B; MIC=minimum inhibitory concentration; NA=not applicable; NR=not reported

a The number in parenthesis represents the total sample of the study, which included other fungal species besides *C. albicans*.

In a cross-sectional study developed by Badiee et al.^[18]^, out of 31 patients with suspected infective endocarditis, 11 had fungal etiology (*Aspergillus* spp. and *C. albicans*). Out of the 11, only two were by *C. albicans*, both intravenous drug users and with fever as a common symptom. They were surgically treated in association with AMB for a minimum of 6 weeks. After treatment, one of the two patients remained with positive blood culture, and both presented positive histopathology. There was no report of death, and after the 12-month follow-up period, they were clinically well.

Analyzing the mean minimum inhibitory concentration (MIC) in both *C. albicans* infected patients, AMB level (0.375 µ/mL) was lower than voriconazole (0.3825 µ/mL), itraconazole (0.75 µ/mL), ketoconazole (1.25 µ/mL) and fluconazole (24 µ/mL), demonstrating a higher sensitivity profile of the agent to AMB treatment compared to these antifungals, but was higher than that of posacoazole (0.079 µ/mL) and caspofungin (0.024 µ/mL).

In another study, nine adults with prosthetic valve endocarditis seen between 1985 and 1996 were infected by *C. albicans*^[19]^. The medical records were evaluated, with emphasis on comorbidities and associated risk factors, microbiological information, treatment modalities and clinical features. The therapy of choice was the association between AMB and valve replacement surgery. They reported only one death, and it was due to surgical complication after a laparotomy and resection of the superior mesenteric artery, portion of the small intestine and spleen after acute abdomen secondary to an aneurysm.

Out of the three patients reported by Flanagan et al.^[20]^, two had *C. albicans* endocarditis. Endocarditis was evaluated by echocardiography and *C. albicans* was isolated by three peripheral blood samples. The first case progressed with operative site necrosis and osteomyelitis involving tibia and fibula, submitted to lower limb amputation surgery, and the patient died. Initially, the AMB MIC was 0.3 *versus* 1.0 for fluconazole, suggesting a higher sensitivity to AMB. In the second case, the patient started treatment with fluconazole for four weeks, then starting oral AMB in combination with oral flucytosine associated with amphotericin B in colloidal dispersion (amphocil), replacing fluconazole. The patient died two weeks later due to heart failure. Blood MIC was <0.25 for AMB and 1.0 for fluconazole.

Two cases of infants (2 and 3 months of age) with endocarditis after congenital heart surgery were presented by Stripeli et al.^[13]^. Both patients received AMB treatment without surgical intervention. Endocarditis was evaluated by echocardiogram and *C. albicans* was isolated through a blood sample. One patient started the treatment with AMB (1 mg/kg/day). Fluconazole (8 mg/kg/day, then 13 mg/kg/day) was included on the 10^th^ day, using this combination for 14 days. After that, AMB was replaced by liposomal amphotericin B (L-AMB) (5 mg/kg/day). With the new medication, blood collection was normal after 4 days, and vegetation in the tricuspid valve disappeared after 28 days. The other patient received L-AMB (5 mg/kg/day) for 7 weeks, with no side effects detected, and subsequent use of fluconazole (5 mg/kg/day) for six months, showing clinical improvement.

Karatza et al.^[21]^ presented other two neonatal pediatric patients born with very low birth weight (<1500 g). The first was female, born at 28 weeks gestation and 1,270 g; and the second, male, 29 weeks and 1,280 g. Endocarditis was evaluated by echocardiogram and *C. albicans* was isolated by a blood sample. Clinical improvement was measured by the presence or absence of candidemia through a blood culture. In the first case, *C. albican*s was isolated in blood culture on day 20, and ampicillin and gentamicin were replaced by L-AMB (5 mg/kg/day), and after six days with persistence of candidemia, caspofungin (1 mg/kg/day and after two days 2 mg/kg/day) was started. On day 40, with persistence of candidemia, caspofungin was replaced by fluconazole (6 mg/kg/day), with improvement in blood culture 72 hours after new therapy. In the second case, L-AMB was started after blood culture results on day 18, and on day 23 was associated with caspofungin. On day 47, caspofungin was replaced by fluconazole, with improvement five days after the start of new therapy. Both patients used the same doses.

## DISCUSSION

Fungal endocarditis represents a low percentage of total endocarditis. However, increases in the number of cases have been described. This is a result of the increase of immunocompromised patients, valve prosthesis surgery, pacemakers implantation, diffusion of central lines and broad-spectrum antibiotic therapy^[7,22,23]^. Badiee et al.^[18]^described the incidence of infective endocarditis after surgical procedures.

Despite being an uncommon condition, FE has a mortality rate >50%, according to the 2015 ESC Guidelines for the management of infective endocarditis^[24]^. In our review, the average mortality rate found in five studies was around 18%. The incidence of FE now comprises 1-10% of all etiological agents isolated in patients with infective endocarditis^[18]^. Within this subgroup of endocarditis, 50-70% are due to *Candida* species, with *C. albicans* being the most common^[4,22]^. *Candida* endocarditis is, therefore, a rare entity that requires special attention due of its high morbidity and mortality^[22]^.

Endocarditis should be suspected in cases when blood cultures are persistently positive, when a patient with candidemia has persistent fever despite appropriate treatment, or when a new heart murmur, heart failure, or embolic phenomenon occur in the setting of candidemia^[25,26]^. Beyond that, clinical features are very important: one of the studies analyzed in our systematic review showed that a patient’s death occurred after treatment discontinuation based on a transthoracic echocardiogram negative for endocarditis^[20]^.

AMB may not effectively penetrate fungal vegetations. The use of a concomitant second agent, such as flucytosine, is recommended, since the association of drugs may potentiate the resolution of fungal vegetations. AMB plus fluconazole have previously been reported to be effective in both native and prosthetic valve *Candida* endocarditis^[21]^.

Medical therapy of *Candida* endocarditis has occasionally been curative, but the optimal therapy for both native and prosthetic valve endocarditis in adults is a combination of valve replacement and a long course of antifungal therapy, based on different studies. Because *Candida* endocarditis has a propensity to relapse months to years later, follow-up should be maintained for several years after treatment^[27]^.

The 2015 Infectious Diseases Society of America guidelines for the management of *Candida* endocarditis state that native valve and prosthetic valve infections should be managed with surgical replacement of the infected valve^[27,28]^. The recommended antifungal therapy for initial therapy is lipid formulation of AMB, 3-5 mg/kg daily, with or without flucytosine, 25 mg/kg 4 times daily, or high-dose echinocandin. In pacemaker infections, implantable cardiac defibrillator and ventricular assist device, the entire device should be removed as soon as possible, considering the risk of causing embolism^[1]^.

According to Noguchi et al.^[14]^, fungal endocarditis is considered an absolute indication for surgery, but is not always possible to remove the infected valve. The high risk of intraoperative mortality ruled out the patient as a surgical candidate. Furthermore, in their study, because the patient suffered clinically insignificant embolic complication and had no vegetation or paravalvular leakage, they did not insist on removing the valve^[29,30]^. Lejko-Zupanc et al.^[12]^ defend that in selected cases, particularly those with few complications or minor vegetation, clinical treatment should be attempted first.

Supporting this recommendation, Rivoisy et al.^[15]^ showed that their multivariate analysis indicated two factors independently associated with lower odds of surgery success: older age and presentation with cardiac failure. In one third of cases, the decision not to operate was motivated by the estimated low severity of the endocarditis, which indicates that surgeons do not systematically follow current guidelines. Importantly, the six-month mortality outcomes in patients not operated were similar to those in patients who underwent surgery^[15]^.

Lefort et al.^[31]^ showed that among 33 cases, the mortality rate was similar regardless of whether surgery was performed or not and any difference was found in prognosis according to the management of *Candida* endocarditis: medical therapy alone *versus* combined with surgery. However, their results suggest that early cardiac surgery during CE should always be attempted, and only patients with very poor medical status might not be operated. For the latter patients, definitive antifungal therapy may be considered.

### Limitations

The data about antifungal therapy in *Candida* FE were very heterogeneous considering differences between drug doses and combination, follow-up period, and patient age. Considering the rarity of *C. albicans* endocarditis, the number of studies was low, as expected.

Our results were based on observational studies comprised by a small sample. To date, no randomized clinical trial evaluated the efficacy and safety of AMB in the context of FE by *C. albican*s. Some guidelines recommend AMB as first-line therapy based on a limited experience with small case series, which represents a very low quality of evidence.

We found as a difficult point the fact that is hard finding scientific evidence to help physicians make clinical decisions, especially in therapeutic terms. We consider that compiling the available information beyond observational studies and case series can help us guide a better pharmacological approach to existing cases.

## CONCLUSION

From our research, AMB seemed to be an important therapeutic option for *C. albicans* endocarditis, decreasing mortality and increasing the survival rate, and presenting a better response confirmed by laboratory and imaging exams.

**Table:** 

Authors' roles & responsibilities	
LSB	Substantial contributions to the conception or design of the work; or the acquisition, analysis or interpretation of data for the work; drafting the work or revising it critically for important intellectual content; final approval of the version to be published
JAS	Substantial contributions to the conception or design of the work; or the acquisition, analysis or interpretation of data for the work; drafting the work or revising it critically for important intellectual content; final approval of the version to be published
MAOSV	Substantial contributions to the conception or design of the work; or the acquisition, analysis or interpretation of data for the work; drafting the work or revising it critically for important intellectual content; final approval of the version to be published
SGL	Substantial contributions to the conception or design of the work; or the acquisition, analysis or interpretation of data for the work; drafting the work or revising it critically for important intellectual content; final approval of the version to be published
AVCM	Substantial contributions to the conception or design of the work; or the acquisition, analysis or interpretation of data for the work; drafting the work or revising it critically for important intellectual content; final approval of the version to be published
MBJ	Substantial contributions to the conception or design of the work; or the acquisition, analysis or interpretation of data for the work; final approval of the version to be published
